# Enhanced proactive control under stress: divergent neural dynamics of social vs. Monetary rewards in table tennis athletes

**DOI:** 10.3389/fspor.2025.1591411

**Published:** 2025-11-19

**Authors:** Lin Xu, Ziyi Peng, Jie Lian, Yongcong Shao, Yuefang Dong, Weiwei Fu

**Affiliations:** 1 School of Psychology, Beijing Sport University, Beijing, China; 2 Mental Health Education Center, Huzhou University, Huzhou, China; 3 Laboratory of Sports Stress and Adaptation of General Administration of Sport, Beijing, China; 4 School of Biomedical Engineering (Suzhou), Division of Life Sciences and Medicine, University of Sciences and Technology of China, Hefei, China; 5 Suzhou Institute of Biomedical Engineering and Technology, Chinese Academy of Sciences, Suzhou, China

**Keywords:** acute stress, proactive control, reward, table tennis athletes, event-related potential

## Abstract

**Background:**

In high-stress competitive sports, athletes rely on proactive control, a core process that maintains goal-relevant information during anticipated conflicts, which is modulated by motivational rewards. While prior studies have established reward's general benefits on cognitive performance, it remains unclear whether monetary (extrinsic) and social (intrinsic) rewards mitigate stress effects through divergent neurocognitive pathways.

**Methods:**

Twenty-three male table tennis athletes (age: 20.435 ± 1.903 years) performed a reward-modified AX-CPT task following acute stress induction via the Maastricht Acute Stress Test (MAST). Proactive control was assessed through behavioral indices (reaction time, error rate) and event-related potentials [ERPs: Cue-P2, Cue-P3, P2, P3b, Contingent Negative Variation (CNV)], capturing neural dynamics of reward anticipation and sustained attention.

**Results:**

Both reward types enhanced proactive control, with social rewards eliciting larger P3b amplitudes than monetary rewards during target processing, reflecting greater sustained attentional engagement, which correlated with their superior behavioral performance. Monetary rewards augmented early attention similar to social rewards, while CNV amplitudes indicated sustained preparatory attention under social rewards.

**Conclusion:**

Monetary and social rewards recruit distinct neurocognitive pathways to enhance athletes' proactive control under stress: monetary rewards prioritize early attentional engagement, whereas social rewards optimize sustained cognitive resource allocation via intrinsic motivation. These findings unveil the electrophysiological basis of reward-contingent stress resilience, advancing dual-mechanism models of cognitive control.

## 1 Introduction

Becoming an elite athlete is a challenging endeavor. For decades, society, the media, and sports institutions have predominantly emphasized athletes' resilience and positive portrayals, often resulting in a reluctance to display vulnerability or to actively seek support. Although the pursuit of the Olympic motto “Faster, Higher, Stronger, Together” is widely celebrated, it is equally critical to acknowledge the complex and multidimensional stressors that athletes encounter within the hierarchical structure of competitive sports (1, 2). These stressors span multiple domains, including training, competition, interpersonal relationships, career transitions, and social evaluations. Specific sources of stress include excessive training loads and chronic fatigue (3), pre-competition anxiety (4, 5), unanticipated disruptions during events, and the frustration associated with poor performance or defeat (6). Interpersonal conflicts with coaches or teammates (7), uncertain career prospects and identity reconstruction following athletic retirement (8), as well as heightened public expectations and persistent media scrutiny (9), also contribute substantially to the overall stress burden.

The ongoing influence of multidimensional stressors affects athletes' physiological states in the short term and may chronically accumulate over time. This accumulation has the potential to impair critical cognitive functions, particularly cognitive control. Cognitive control plays a pivotal role in maintaining goal-relevant information in working memory, suppressing irrelevant distractions, and preparing for upcoming tasks, thereby rendering it essential in competitive, fast-paced sporting environments. The Dual Mechanisms of Control theory (DMC) conceptualizes cognitive control as comprising two complementary control modes: proactive control and reactive control (10). Proactive control functions as an early selection mechanism, operating during the preparatory phase of a task. It entails the selective attention to task-relevant cues, active maintenance of this information in working memory, and formulation of a response plan based on anticipated task demands. Through this process, proactive control enhances anticipatory readiness and suppresses potential conflicts before they emerge. In contrast, reactive control serves as a late correction mechanism that is recruited during response execution. It involves the flexible use of immediately available task information to resolve conflicts, and when necessary, retrieves previously encoded cues to adjust ongoing responses and correct errors. Effective cognitive performance in dynamic environments relies on the flexible coordination between these two modes. Given the critical role of anticipatory preparation in athletic performance, the present study focuses on how acute stress modulates proactive control, and whether different types of rewards can mitigate these impairments.

Optimal proactive control is essential for athletes, as it serves as the cognitive foundation for maintaining stable attention, resisting distractions, and making rapid decisions during competitions (11). However, this key mechanism often exhibits significant vulnerability under stress conditions (12). Research indicates that acute stress can weaken prefrontal cortex function through dual neurochemical pathways (13–16), thereby impairing proactive control and leading to deficits in goal representation and attentional regulation (17). During this process, athletes may experience issues such as diminished attention (18), impaired working memory (19, 20), and diminished self-regulation capacity (21), all of which can significantly impair athletic performance. Therefore, exploring methods to mitigate the adverse effects of acute stress and strengthen the robustness of proactive control mechanisms is crucial for optimizing athletes' competitive performance.

In the neuroregulatory response to stress, the hypothalamic-pituitary-adrenal (HPA) axis functions as a core neuroendocrine system, whose activity is regulated by a multi-level reward system. Research indicates that the ventromedial prefrontal cortex exerts bidirectional regulation of the HPA axis by modulating the basolateral amygdala-nucleus accumbens circuitry, in conjunction with dopamine and endogenous opioid systems (22–25). This regulatory mechanism antagonizes the excessive activation of the HPA axis under stress, thereby effectively alleviating acute stress responses (26). In addition to lowering stress levels, rewards can enhance proactive control, facilitating the adoption of more goal-directed cognitive processing modes, thereby improving attention maintenance and decision-making capabilities in stressful situations (27, 28). For example, Chiew and Braver (11) demonstrated through high-resolution pupil measurements that rewards significantly amplify pupil dilation responses, indicating that individuals can maintain higher levels of goal-directed attention when driven by rewards, thereby optimizing cognitive control.

Monetary rewards are widely recognized for their ability to promote goal-directed behavior by enhancing extrinsic motivation (29). In the context of competitive sports, such rewards are prevalent: professional athletes often receive performance-based bonuses in elite competitions, while collegiate athletes may obtain scholarships in recognition of their athletic excellence. When implemented appropriately, monetary incentives can effectively improve athletic performance. However, an overreliance on extrinsic rewards has been associated with reductions in athletes' intrinsic motivation, especially under high-pressure and competitive conditions (30, 31).

Beyond monetary incentives, individuals also respond to a range of social rewards, such as praise, adherence to social norms, the pursuit of self-actualization, and the desire for reciprocity (32–34). In athletic settings, social rewards, such as coach approval and a sense of team belonging. play a critical role in promoting intrinsic motivation. According to Self-Determination Theory (SDT), intrinsic motivation is supported when three fundamental psychological needs are met: autonomy, competence, and relatedness (35). Unlike monetary rewards, which primarily enhance extrinsic motivation and short-term behavioral efficiency under stress (36, 37), social rewards contribute to long-term well-being by fostering sustained intrinsic engagement and attentional control (38–40). Despite these theoretical and empirical insights, limited research has directly compared how monetary and social rewards differentially influence the neurocognitive mechanisms underlying proactive control in high-stress athletic environments. Such mechanisms are critical for regulating attention, maintaining goal-relevant information, and adapting to pressure.

In light of the need to clarify the dynamic interplay between social rewards and proactive control under conditions of acute stress, this study focuses on table tennis players as the focal population. Existing research indicates that long-term, systematic professional training not only promotes the development of complex cognitive functions, such as attention allocation, motor control, and decision-making, but also induces adaptive changes in brain function and structure, which are closely linked to neural plasticity (41–43). Open-skill sports require athletes to rapidly process external cues, anticipate opponents' actions, and respond flexibly and promptly in dynamic and unpredictable environments. Long-term engagement in such sports typically enhances sensitivity to external stimuli, cognitive flexibility, inhibitory control, and rapid decision-making (44, 45). As a quintessential open-skill sport, table tennis is characterized by a small ball size, high velocity, strong spin, and diverse shot variability (46). It requires athletes not only to execute a wide range of shots but also to rapidly respond to opponents' unpredictable returns, placing exceptionally high demands on perceptual speed, decision-making, and proactive control. Furthermore, the binary competitive format of table tennis offers a relatively simplified interactional context, minimizing confounding variables such as team-based tactics and thereby enhancing experimental internal validity. Importantly, real-time feedback from opponents during matches functions both as implicit social rewards and as direct performance-modulating signals. This ecological characteristic makes table tennis players an ideal expert model for investigating the relationship between reward processing and cognitive control mechanisms under stress.

To capture the temporal dynamics of cognitive control under such conditions, the present study adopts high-temporal-resolution event-related potential (ERP) technique offers critical neurophysiological evidence (47). This study identified specific ERP components—including Cue-P2, Cue-P3, P2, P3b, and CNV—as neural indices of proactive control, enabling systematic investigation of distinct cognitive processing stages. Appearing approximately 200 ms following a cue, the Cue-P2 component reflects early perceptual processing and indicates attention allocation to task-relevant stimuli (48). An increased Cue-P2 amplitude suggests heightened attentional engagement. The Cue-P3 component, which occurs between 300 and 600 ms following the cue, is associated with the allocation of cognitive resources and contextual evaluation in anticipation of rewards, where an increased amplitude reflects enhanced processing and valuation of reward anticipation (49, 50). Similarly, the P2 and P3b components represent sustained attention and memory processing; increased amplitudes indicate greater cognitive resource allocation to reward-related stimuli (51, 52). The contingent negative variation (CNV), a slow-wave component that precedes a target stimulus, signifies expectancy and preparatory attention. An increased CNV amplitude indicates heightened proactive control (53).

This study examines how images of cheering spectators, which constitute a critical social rewards mechanism in athletic contexts, differ from monetary rewards in strengthening proactive control of table tennis players exposed to acute stress. By examining ERP responses, we aim to clarify the effects of both reward types on stress management and cognitive control. We hypothesize that both monetary and social rewards will attenuate stress-induced deficits in proactive control, as evidenced by improved behavioral indices (higher Proactive Behavioral Index, PBI) and enhanced neural markers of goal maintenance (larger P3b and CNV amplitudes). Critically, social rewards are predicted to exert a stronger effect than monetary rewards, particularly on P3b amplitudes during target evaluation and CNV amplitudes during response preparation. This divergence stems from the inherent capacity of social rewards to fulfill core psychological needs (autonomy, competence, relatedness) outlined in Self-Determination Theory, which amplifies intrinsic motivation and upregulates ventromedial prefrontal cortex engagement.

## 2 Materials and methods

### 2.1 Participants

In this study, a within-subjects experimental design was employed, consisting of a 2 (reward type: monetary rewards/social rewards) × 4 (stimulus type: AX/AY/BX/BY) factorial structure. Sample size calculations were performed using G*Power 3.1.9 with the following parameters: effect size *f* = 0.25, *α* = 0.05, power = 0.8, number of groups = 1; number of measurements = 8. The analysis indicated that a minimum sample size of 16 participants is required to detect the anticipated effects. In females, HPA axis activity is dynamically modulated by cyclic fluctuations of ovarian hormones, resulting in significant variations in cortisol and adrenocorticotropic hormone (ACTH) levels across menstrual cycle phases (54). In contrast, male HPA axis activity is primarily regulated by external stressors rather than endogenous hormonal cycles, exhibiting smaller baseline fluctuations (55–57). To mitigate confounding effects of sex differences and menstrual cycle variability on stress responses, and in accordance with established participant selection criteria from prior studies (21, 58, 59) combined with sample size calculations, this study enrolled 23 male table tennis athletes (age: 20.435 ± 1.903 years) as experimental participants (Table 1).

**Table 1:** Demographic information of participants (*M* ± *SD*).

Variables	Table tennis athletes
Age, years	20.435 ± 1.903
Gender	Male
BMI, kg/m^2^	22.197 ± 1.987
Time of table tennis played per week, h	8.261 ± 3.899
Yertiary education, years	2.565 ± 1.502

Participants met the following criteria: voluntary consent to participate; good physical health with normal or corrected vision; at least 3 years of professional training and a national ranking of level two or higher; right-handedness; a Beck's Depression Scale score <14, indicating good mental health; and no history of participation in stress-related experiments. All selected athletes completed the monetary rewards task, with 21 completing the social rewards task and 20 completing both tasks.

### 2.2 Experimental materials and tasks

1. Heart rate: Physiological stress was monitored using a Polar H9 heart monitor. Participants' heart rates were continuously monitored to assess physiological stress markers throughout the sessions.
2. Self-reported stress: Participants rate their stress and tension levels on a 5-point scale from 1 (very relaxed) to 5 (very nervous) (60).
3. Acute stress induction: The MAST task, which integrates a social evaluation cold-pressure task (SECPT) with a mental arithmetic (MA) task, was used to induce stress (61). This entailed controlled task switching and interactions with the experimenters to amplify the stress responses. Task switching was controlled based on participants' instructions to augment their sense of uncertainty. During the SECPT, participants were required to immerse their left hand in ice water at 0–2 °C for a maximum of 90 s at a time. Opposite-sex experimenters gazed at the participants during the task to amplify psychological stress and recorded facial expressions. Following the social appraisal cold pressurization task, participants engaged in an MA task, subtracting 17 from 2,043 sequentially. Errors required a mandatory restart of the calculation, lasting at least 45 s.
4. The reward-modified AX-Continuous Performance (AX-CPT) Task was employed to assess the impact of monetary and social rewards on participants' proactive control capabilities. This task integrates two types of reward stimuli: monetary rewards, symbolized by coin images and piggy bank feedback, and social rewards, represented by emoticon cues and audience applause feedback. The selection of these cues was grounded in established paradigms of reward processing. Previous studies have shown that monetary images effectively signal extrinsic incentives and can be directly linked to performance-based gains (29, 62). In line with conditioning frameworks, coin images acquire incentive salience through repeated association with subsequent monetary outcomes, thereby serving as an intuitive proxy for real-world financial incentives (63). To ensure consistency in visual form, coin images were chosen over banknotes to match the circular shape of the emoji stimuli, thus controlling for geometric confounds. For social rewards, we adopted emoticon images, which have been validated as effective proxies for social feedback. Smiling faces, whether real or stylized, reliably activate social cognitive mechanism, and are perceived as rewarding stimuli (64, 65). Emoticons combine the socio-emotional significance of facial expressions with the simplicity of symbolic shapes, allowing for efficient visual processing while still engaging neural systems associated with social motivation.

To ensure comparability between the intensity of monetary and social rewards, a subjective value assessment of the reward materials was conducted prior to the formal experiment. Following the logic of psychophysical methods, the social reward image (a smiling face) was defined as the standard stimulus. Participants were then asked to evaluate which monetary amount elicited a comparable level of pleasure and motivational drive. They were presented with three candidate values (1 RMB, 5 RMB, and 10 RMB) and selected the amount they perceived as most equivalent to the social rewards stimulus. Based on the average of participants' choices in the pilot assessment, the 1 RMB denomination was identified as the optimal match. Accordingly, in the main experiment, the coin image represented a reward of 1 RMB for each correct trial, with the accumulated amount displayed in a piggy bank. In order to enhance ecological validity, participants were informed that the total accumulated monetary rewards would be exchanged at a 1:1 rate and paid out in real currency at the conclusion of the experiment (up to 300 RMB if all trials in the monetary rewards condition were answered correctly). The smiley face indicated forthcoming social approval in the form of audience applause. This design ensured that both reward types were standardized in intensity and directly tied to performance outcomes.

**Figure 1:**
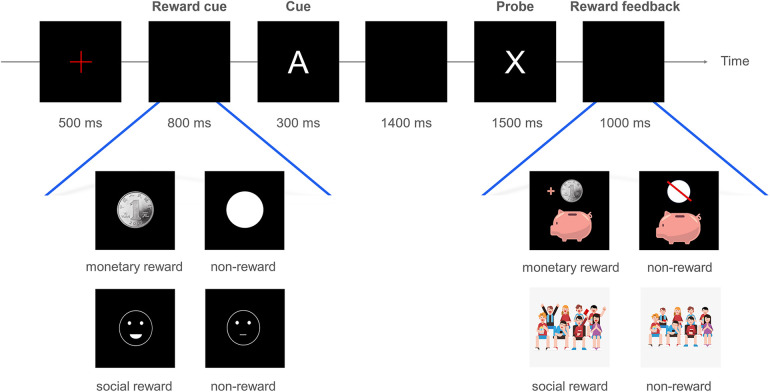
Flowchart of the AX-CPT task with monetary and social rewards. For the monetary rewards condition, reward cues are represented by coin images, and non-reward cues by white circles; correct responses result in a visual of coins being deposited into a piggy bank. In the social rewards condition, reward cues are shown as smiling faces, non-reward cues as neutral expressions; correct responses lead to feedback of audience cheers and applause.

Each trial in the AX-CPT involves presenting a cue letter, “A” or another letter, followed by a probe letter, “X” or a different one. The task consists of four trial types—AX, AY, BX, and BY. “B” signifies any letter except A, X, Y, or K, while “Y” denotes any letter except A, B, X, or K. Participants must press the “S” key for the target AX trials and the “K” key for all other combinations. The experiment was divided into two tasks, each focusing on either monetary or social rewards. Each task comprised four blocks of 150 trials. The trials were randomly sequenced in a 7:1:1:1 ratio of AX to AY, BX, and BY, with an equal number of reward and control condition trials. Participants had to maintain an accuracy rate above 85% to ensure progression. The task sequence was as follows: a fixation point displayed for 500 ms, followed by an 800 ms reward cue, a 300 ms cue letter, a 1,400 ms blank interval, and finally, a 1,500 ms probe letter presentation. Responses were required within 1,500 ms after the probe letter appeared (see Figure 1 for a visual representation).

### 2.3 Experimental procedures

**Figure 2:**
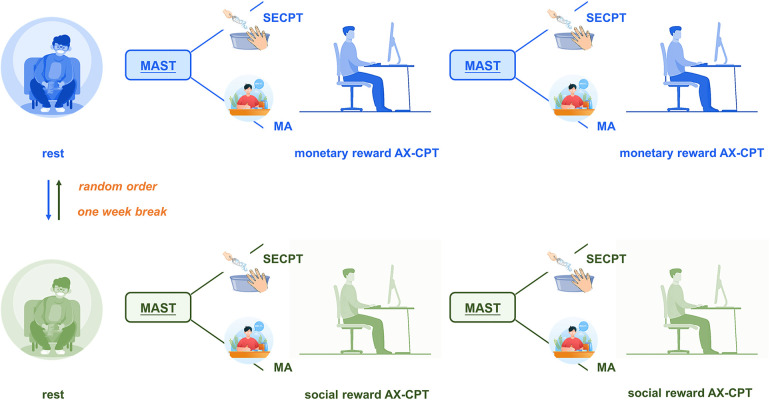
Overview of the experimental procedure for monetary and social rewards tasks. Participants underwent monetary and social rewards tasks in a randomized order across two sessions spaced one week apart. The experimental protocol involved completing the first two blocks of the reward-based AX-CPT task post-stress induction, followed by a second acute stress induction to maintain the stress state and concluding with the final two blocks of the task.

After participants met the inclusion criteria, they were scheduled for two sessions spaced one week apart, each dedicated to either the monetary or social rewards task (Figure 2). The order of the two tasks was randomly assigned across participants to avoid systematic sequence effects. The one-week interval between sessions was deliberately chosen to minimize potential carryover and practice effects, ensuring that performance in one condition did not influence the other. Pre-experimental instructions advised participants to avoid intense exercise, alcohol, sugar, and caffeinated beverages. Upon arriving at the laboratory, participants were briefed, gave informed consent, and were equipped with heart rate and EEG monitors. They underwent practice tasks to familiarize themselves with the procedure.

Each session commenced with stress induction using the Maastricht Acute Stress Task (MAST). Immediately following this induction, participants performed the AX-CPT reward tasks, ensuring that stress endured into the primary task phase. Participants completed two blocks of the cognitive task following the first stress induction, then underwent a second stress induction before continuing with the remaining two blocks. This design choice was guided by prior research showing that stress responses, as measured by salivary cortisol, typically peak about 25 min after induction and persist for approximately 35–60 min (66, 67). By introducing a second stress induction midway through the experiment, we ensured that participants remained in a high-stress state throughout the entire reward-modified AX-CPT task, thus allowing for a more reliable assessment of task performance under sustained acute stress. To systematically investigate the effects of acute stress, we recorded participants' heart rate and self-reported stress levels at five key time points: baseline (t1), immediately after the first acute stress induction (t2), following completion of the first AX-CPT task (t3), after the second acute stress induction (t4), and after completing the second AX-CPT task (t5). This sequence allowed us to capture the dynamic process of stress responses and subsequent recovery. The study protocol received approval from the Beijing Sport University Ethics Committee (No. 2023313H) and adhered to the Declaration of Helsinki.

### 2.4 Data acquisition and analysis

#### 2.4.1 Behavioral data analysis

Building on prior research (68, 69), a direct index, the proactive control index (PBI), was computed to reflect the cognitive control tradeoff in the behavioral analyses. This index is calculated using the response times or error rates from AY and BX trials according to the formula PBI = (AY − BX)/(AY + BX), illustrating the relative interference balance between these trials and individuals' propensity toward proactive control. The PBI ranges from −1 to +1, with a higher value indicating a stronger inclination for individuals to employ proactive control strategies. Paired-sample *t*-tests were conducted using SPSS software (version 27.0) to compare the PBI for response time and error rate between different conditions: monetary rewards (yes vs. no), social rewards (yes vs. no), and reward types (monetary vs. social rewards).

#### 2.4.2 ERP data acquisition

The data experiment obtained EEG data using a 32-channel Neuroscan system (Compumedics Neuroscan, Charlotte, NC, USA) with a standard EEG cap following a 10–5 system. Vertical electrooculograms were captured with electrodes positioned 2 cm below the left eye, while horizontal electrooculograms were captured at the lateral edge of the right eye. Channel resistances were maintained below 5 K*Ω* for all channels during online ERP activity collection. Offline EEG data analysis was conducted using the EEGLAB toolbox (70) in MATLAB 2018b (MathWorks, Natick, MA, USA). In the offline preprocessing, the EEG data were downsampled to 500 Hz. Subsequently, the downsampled data underwent a band-pass filter between 0.1 and 30 Hz. After filtering, the data were segmented into epochs and baseline-corrected. Bad segments were excluded, and faulty channels and epochs were interpolated. Independent Component Analysis (ICA) was applied to correct for artifacts related to eye movements. Any residual artifacts were excluded with a threshold set at ±100 μV. The average scalp signal was used for re-referencing.

#### 2.4.3 ERP data analysis

The selection of electrode sites was based on a comprehensive review and synthesis of the existing relevant literature (71, 72), combined with direct observational results from the ERP data.

##### 2.4.3.1 Reward anticipation-related ERP components

The reward cue was time-locked from 200 ms before its presentation to 800 ms after the presentation of the reward cue, with the 200 ms before the presentation of the reward cue used as the baseline correction. Cue-P2 component (time window: 200–300 ms): The amplitude was computed using the mean values of the Fz and FCz electrodes. Cue-P3 component (time window: 320–420 ms, social rewards time window: 340–420 ms): The amplitude was computed using the mean values of the P3, Pz, and P4 electrodes.

ERP components associated with reward expectations were examined using one-way repeated-measures ANOVAs for the Cue-P2 and Cue-P3 components. Analyses were conducted for monetary rewards (yes vs. no), social rewards (yes vs. no), and reward types (monetary vs. social rewards) using SPSS 27.0 software.

##### 2.4.3.2 Proactive control-related ERP components

The cue letter was time-locked from 1,000 ms before its presentation to 1,700 ms after its presentation, with the 1,000–800 ms preceding cue letter presentation used for baseline correction. P2 component (time window: 160–300 ms): For monetary and social rewards, the P2 amplitude was computed by averaging the F3, Fz, F4, FC3, FCz, and FC4 electrodes. The Fz, FCz, and Cz electrodes were averaged for the reward type. P3b component (time window: 400–600 ms): For monetary and social rewards, the P3b amplitude was computed by averaging the FC3, FCz, FC4, Cz, CPz, and Pz electrodes. The C3, Cz, and CPz electrodes were averaged for the reward type. CNV component (time window: 1,500–1,700 ms): The CNV amplitude was calculated by averaging the FCz, Cz, and CPz electrodes for monetary rewards and reward types. For social rewards, the FCz and Cz electrodes were averaged.

ERP components associated with proactive control were analyzed using three 2 × 2 repeated-measures ANOVAs for the P2, P3b, and CNV components. The factors considered in these analyses were as follows: monetary rewards (yes vs. no) × cue type (A vs. B); social rewards (yes vs. no) × cue type (A vs. B); reward type (monetary rewards vs. social rewards) × cue type (A vs. B). In cases where the assumption of sphericity was violated, multivariate analyses were employed, and the results were adjusted for multiple comparisons of all main effects using the LSD method.

## 3 Results

### 3.1 Acute stress manipulation examination

Participants were exposed to acute stress twice during each monetary and social rewards experiment. Heart rate and self-reported stress perception were statistically analyzed before and after each acute stress manipulation. The Supplementary Material includes detailed results demonstrating significant increases in heart rate and self-reported stress perception scores among table tennis athletes following acute stress manipulation compared to pre-acute stress conditions.

### 3.2 Behavioral performance

#### 3.2.1 Monetary rewards PBI

**Figure 3:**
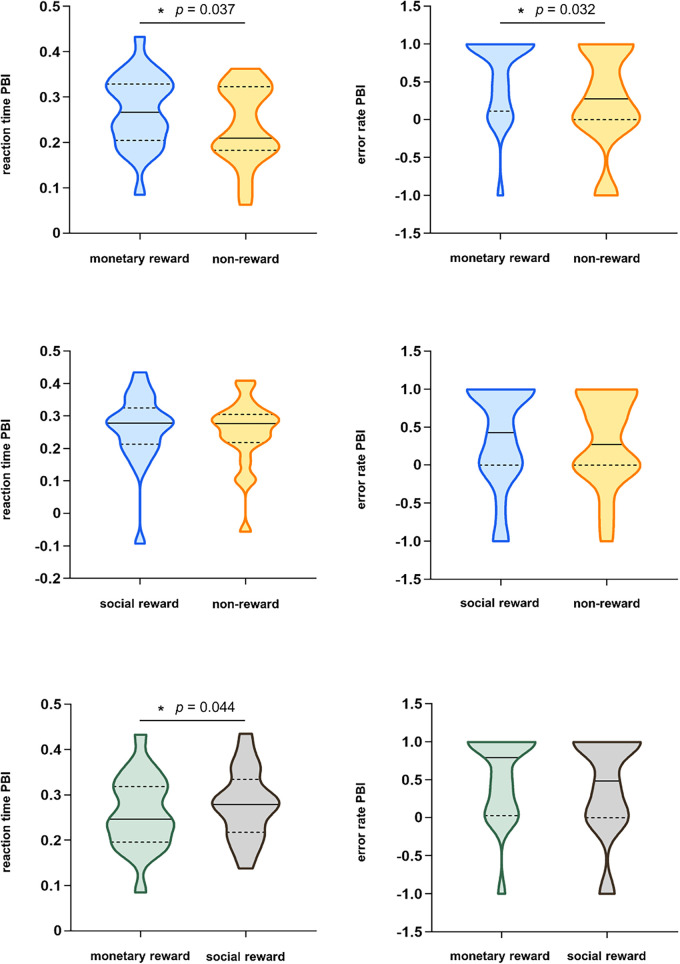
Reaction time and error rate proactive control index (PBI) of all conditions (*M* ± SD). *p < 0.05.

The paired-samples *t*-tests results revealed that under acute stress, the table tennis athlete's PBI was significantly higher in the monetary rewards condition for both reaction time (*M* = 0.270, *SD* = 0.080) and error rate (*M* = 0.607, *SD* = 0.541) compared to the no-reward condition (reaction time: *M* = 0.237, *SD* = 0.085, *t*(22) = 2.215, *p* = 0.037, Cohen's *d* = 0.462; error rate: *M* = 0.262, *SD* = 0.652, *t*(22) = 2.287, *p* = 0.032, Cohen's *d* = 0.477. Remarkably, anticipating a monetary rewards induced stronger proactive control in table tennis athletes under acute stress than under no-reward anticipation conditions (Figure 3).

#### 3.2.2 Social rewards PBI

The paired-sample *t*-test results on the social rewards PBI, based on the reaction time following the acute stress induction, revealed a nonsignificant difference between the social rewards condition (*M* = 0.261, *SD* = 0.110) and the no-reward condition (*M* = 0.249, *SD* = 0.105), *t*(20) = 0.675, *p* = 0.507, Cohen's *d* = 0.147). Similarly, the paired-samples *t*-test for social rewards PBI based on error rates showed a nonsignificant difference between the social rewards condition (*M* = 0.378, *SD* = 0.670) and the no-reward condition (*M* = 0.311, *SD* = 0.605), *t*(20) = 0.334, *p* = 0.742, Cohen's *d* = 0.073 (Figure 3).

#### 3.2.3 Monetary rewards PBI vs. social rewards PBI

Paired-sample *t*-tests revealed that following acute stress induction, the table tennis athletes' reaction time PBI was significantly higher in the social rewards condition (*M* = 0.279, *SD* = 0.077) than in the monetary rewards condition (*M* = 0.260, *SD* = 0.082), *t*(19) = 2.157, *p* = 0.044, Cohen's *d* = 0.482. Furthermore, no significant difference was observed in the PBI values based on error rates between the social rewards condition (*M* = 0.423, *SD* = 0.652) and the monetary rewards condition (*M* = 0.548, *SD* = 0.558), *t*(19) = 0.665, *p* = 0.514, Cohen's *d* = 0.149 (Figure 3).

### 3.3 ERP results

#### 3.3.1 Monetary rewards

##### 3.3.1.1 Reward anticipation ERP components

**Figure 4:**
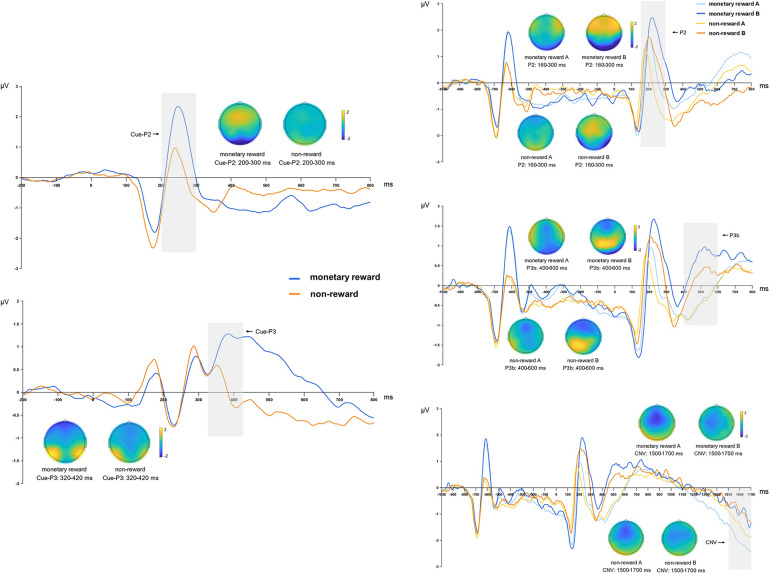
The topographies correspond to average activity in the time windows (gray bar) around the local peaks. Left side: Grand mean ERP amplitude of table tennis athletes on Cue-P2 and Cue-P3 in monetary rewards and non-reward conditions. Right side: Grand mean ERP amplitude of table tennis athletes on P2, P3b and CNV in monetary rewards A/B and non-reward A/B conditions.

The analysis revealed a significant main effect of reward anticipation for both the Cue-P2 [*F*_(1, 22)_ = 31.406, *p* < 0.001, *η*^2^*_p_* = 0.588] and Cue-P3 [*F*_(1, 22)_ = 7.290, *p* = 0.013, *η*^2^*_p_* = 0.249] components. The cue-P2 and cue-P3 amplitudes were notably larger in the monetary rewards anticipation condition than in the no-reward anticipation condition (Table 2, Figure 4).

**Table 2:** Mean and standard deviation of Cue-P2 and Cue-P3 amplitudes across reward comparisons (*M* ± *SD*).

Comparison	Reward condition	Cue-P2 (μV)	Cue-P3 (μV)
Monetary rewards vs. no-reward	Monetary	2.600 ± 1.642	1.687 ± 1.693
	No-reward	1.290 ± 1.089	0.883 ± 0.849
Social rewards vs. no-reward	Social	2.147 ± 1.650	1.839 ± 1.606
	No-reward	1.216 ± 1.318	0.695 ± 1.066
Monetary rewards vs. social rewards	Monetary	2.420 ± 1.511	1.477 ± 1.668
	Social	2.185 ± 1.683	1.825 ± 1.589

##### 3.3.1.2 Proactive control of ERP components

Analysis of the P2 component revealed a significant main effect of monetary rewards [*F*_(1, 22)_ = 5.399, *p* = 0.030, *η*^2^*_p_* = 0.197], indicating that P2 amplitude was notably higher under the monetary rewards condition than the no-reward condition. Additionally, a significant main effect of cue type was observed [*F*_(1, 22)_ = 27.162, *p* < 0.001, *η*^2^*_p_* = 0.552], with cue letter B inducing a significantly larger P2 amplitude than cue letter A. The analysis did not exhibit a significant relationship between monetary rewards and cue type [*F*_(1, 22)_ = 0.311, *p* = 0.582, *η*^2^*_p_* = 0.014]. Further investigation revealed that regardless of the presence (*p* = 0.001) or absence (*p* < 0.001) of a reward, cue letter B consistently elicited a larger P2 amplitude than cue letter A (Table 3, Figure 4).

**Table 3:** Mean and standard deviation of P2, P3b and CNV amplitudes across reward comparisons (*M* ± *SD*).

Comparison	Reward condition	Cue type	P2 (μV)	P3b (μV)	CNV (μV)
Monetary rewards vs. no-reward	Monetary	A	2.186 ± 1.555	0.257 ± 0.929	−2.631 ± 1.915
	No-reward		1.836 ± 1.460	0.176 ± 0.701	−1.981 ± 1.654
	Monetary	B	3.048 ± 2.018	1.748 ± 1.118	−1.814 ± 1.417
	No-reward		2.565 ± 1.400	1.181 ± 1.027	−1.774 ± 1.773
Social rewards vs. no-reward	Social	A	1.827 ± 1.474	0.123 ± 0.785	−3.513 ± 2.224
	No-reward		1.694 ± 1.286	0.249 ± 0.753	−3.162 ± 2.048
	Social	B	3.370 ± 1.597	2.101 ± 1.752	−2.286 ± 1.567
	No-reward		2.350 ± 1.574	1.334 ± 1.205	−2.744 ± 1.820
Monetary rewards vs. social rewards	Monetary	A	1.682 ± 1.716	0.278 ± 1.212	−2.600 ± 1.943
	Social		2.003 ± 1.734	0.297 ± 0.829	−2.679 ± 1.870
	Monetary	B	2.602 ± 2.448	1.662 ± 1.440	−1.879 ± 1.501
	Social		3.602 ± 1.949	2.607 ± 1.564	−1.898 ± 1.450

The P3b component analysis revealed a significant interaction between monetary rewards and cue type [*F*_(1, 22)_ = 4.457, *p* = 0.046, *η*^2^*_p_* = 0.168]. *Post-hoc* analysis revealed that monetary rewards resulted in larger P3b amplitudes in the cue letter B condition than in the no-reward condition (*p* = 0.008). Additionally, irrespective of the reward, the P3b amplitude induced by cue letter B was consistently greater than that induced by cue letter A (*p* < 0.001). The monetary rewards had a significant main effect [*F*_(1, 22)_ = 9.442, *p* = 0.006, *η*^2^*_p_* = 0.300], with the P3b amplitude significantly larger in the monetary rewards condition than in the no-reward condition. The main effect of cue type was significant [*F*_(1, 22)_ = 83.598, *p* < 0.001, *η*^2^*_p_* = 0.792], indicating that cue letter B induced significantly greater P3b amplitude than cue letter A (Table 3, Figure 4).

The analysis of the CNV component revealed a marginally significant main effect of monetary rewards [*F*_(1, 22)_ = 4.028, *p* = 0.057, *η*^2^*_p_* = 0.155], indicating that the monetary rewards condition resulted in larger negative CNV amplitudes. Additionally, the main effect of cue type was significant [*F*_(1, 22)_ = 4.449, *p* = 0.047, *η*^2^*_p_* = 0.168], showing that cue letter A induced larger CNV amplitudes than cue letter B. However, the relationship between monetary rewards and cue type was insignificant [*F*_(1, 22)_ = 2.261, *p* = 0.147, *η*^2^*_p_* = 0.093]. Further analysis revealed that the monetary rewards induced larger CNV amplitudes in the cue letter A condition (*p* = 0.012), while cue letter A induced significantly larger CNV amplitudes than cue letter B in the monetary rewards condition (*p* = 0.029). Additionally, the difference in cue type was not significant in the no-reward condition (*p* = 0.464) (Table 3, Figure 4).

#### 3.3.2 Social rewards

##### 3.3.2.1 Reward anticipation ERP components

**Figure 5:**
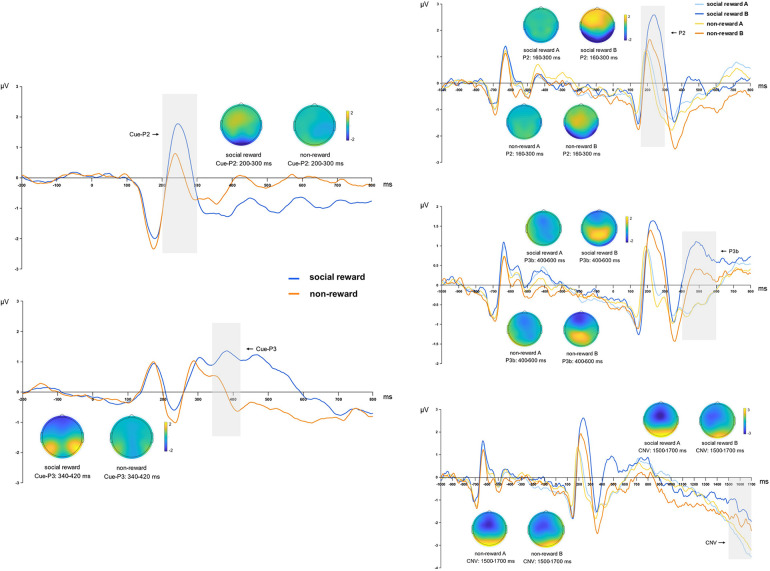
The topographies correspond to average activity in the time windows (gray bar) around the local peaks. Left side: Grand mean ERP amplitude of table tennis athletes on Cue-P2 and Cue-P3 in social rewards and non-reward conditions. Right side: Grand mean ERP amplitude of table tennis athletes on P2, P3b and CNV in social rewards A/B and non-reward A/B conditions.

The analysis revealed a significant main effect of reward anticipation for the Cue-P2 [*F*_(1, 20)_ = 14.145, *p* = 0.001, *η*^2^*_p_* = 0.414] and Cue-P3 [*F*_(1, 20)_ = 17.861, *p* < 0.001, *η*^2^*_p_* = 0.472] components. The cue-P2 and cue-P3 amplitudes were notably larger in the social rewards anticipation condition than in the no-reward anticipation condition (Table 2, Figure 5).

##### 3.3.2.2 Proactive control of ERP components

The P2 components analysis revealed a significant interaction between social rewards and cue type [*F*_(1, 20)_ = 12.592, *p* = 0.002, *η*^2^*_p_* = 0.386]. Simple effects analyses showed that social rewards resulted in larger P2 amplitudes in the cue letter B condition than in the no-reward condition (*p* = 0.001). Moreover, irrespective of the presence of a reward (*p* < 0.001) or no reward (*p* = 0.013), cue letter B consistently induced larger P2 amplitudes than cue letter A. The main effect of social rewards was significant [*F*_(1, 20)_ = 8.636, *p* = 0.008, *η*^2^*_p_* = 0.302], with reward inducing larger P2 amplitudes than no-reward. Additionally, a significant main effect of cue type [*F*_(1, 20)_ = 30.821, *p* < 0.001, *η*^2^*_p_* = 0.606] was noted, indicating that cue letter B elicited significantly larger P2 amplitudes than cue letter A (Table 3, Figure 5).

In the analysis of the P3b component, the interaction between social rewards and cue type was significant [*F*_(1, 20)_ = 12.819, *p* = 0.002, *η*^2^*_p_* = 0.391]. Simple effects analyses revealed that social rewards induced larger P3b amplitudes in the cue letter B condition than in the no-reward condition (*p* = 0.007). Moreover, cue letter B consistently induced larger P3b amplitudes than cue letter A, irrespective of the presence of a reward (*p* < 0.001). The main effect of social rewards was also significant [*F*_(1, 20)_ = 5.511, *p* = 0.029, *η*^2^*_p_* = 0.216], indicating that the P3b amplitude was significantly higher in the social rewards condition than the no-reward condition. Additionally, a significant main effect of cue type was observed [*F*_(1, 20)_ = 42.026, *p* < 0.001, *η*^2^*_p_* = 0.678], with cue letter B inducing significantly greater P3b amplitudes than cue letter A (Table 3, Figure 5).

The CNV components analysis showed a significant interaction between social rewards and cue type [*F*_(1, 20)_ = 6.374, *p* = 0.020, *η*^2^*_p_* = 0.242]. Further simple effects analysis indicated that cue letter A induced larger CNV amplitudes than cue letter B in the social rewards condition (*p* = 0.001). However, the main effect of social rewards was nonsignificant [*F*_(1, 20)_ = 0.105, *p* = 0.749, *η*^2^*_p_* = 0.005]. Conversely, a significant main effect of cue type was observed [*F*_(1, 20)_ = 10.161, *p* = 0.005, *η*^2^*_p_* = 0.337], indicating that cue letter A yielded a significantly larger CNV amplitude than cue letter B (Table 3, Figure 5).

#### 3.3.3 Monetary rewards vs. social rewards

##### 3.3.3.1 Reward anticipation ERP components

**Figure 6:**
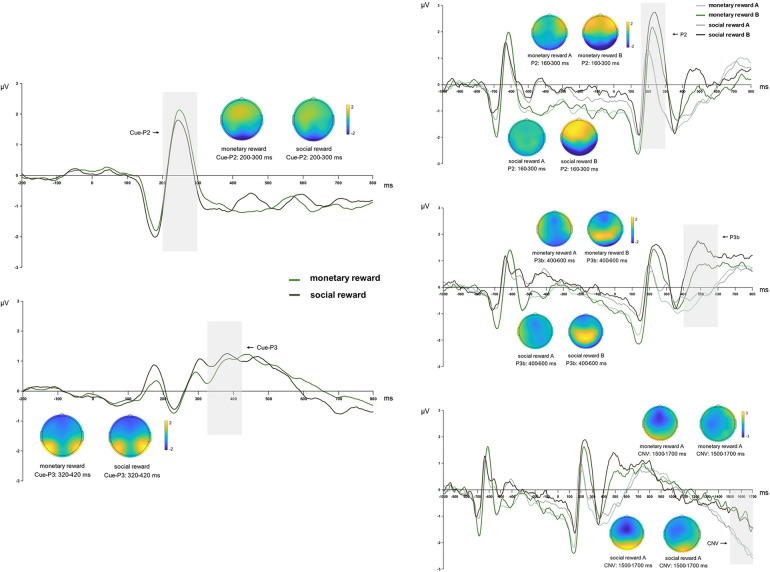
The topographies correspond to average activity in the time windows (gray bar) around the local peaks. Left side: Grand mean ERP amplitude of table tennis athletes on Cue-P2 and Cue-P3 in monetary rewards and social rewards conditions. Right side: Grand mean ERP amplitude of table tennis athletes on P2, P3b and CNV in monetary rewards and social rewards A/B conditions.

The analysis revealed that for the Cue-P2 and Cue-P3 components, the main effect of reward type was not significant [Cue-P2: *F*_(1, 19)_ = 1.094, *p* = 0.309, *η*^2^*_p_* = 0.054; Cue-P3: *F*_(1, 19)_ = 0.701, *p* = 0.413, *η*^2^*_p_* = 0.036]. This indicates no significant difference in component amplitudes between the monetary and social rewards cues (Table 2 and Figure 6).

##### 3.3.3.2 Proactive control ERP components

The P2 components analysis revealed a significant main effect of cue type [*F*_(1, 19)_ = 38.436, *p* < 0.001, *η*^2^*_p_* = 0.669], with cue letter B inducing significantly larger P2 amplitudes than cue letter A. The main effect of reward type was not significant [*F*_(1, 19)_ = 2.374, *p* = 0.140, *η*^2^*_p_* = 0.111], and the relationship between reward type and cue type was also not significant [*F*_(1, 19)_ = 2.806, *p* = 0.110, *η*^2^*_p_* = 0.129]. Further analysis indicated that social rewards induced larger P2 amplitudes (*p* = 0.057) than monetary rewards in the cue letter B condition, with a marginally significant difference. Moreover, monetary rewards (*p* = 0.009) and social rewards (*p* < 0.001) resulted in cue letter B consistently inducing larger P2 amplitudes than cue letter A (Table 3, Figure 6).

The P3b component analysis revealed a significant interaction between reward and cue types [*F*_(1, 19)_ = 4.606, *p* = 0.045, *η*^2^*_p_* = 0.195]. A simple effects analysis showed that social rewards induced larger P3b amplitudes than monetary rewards in the cue letter B condition (*p* = 0.020). Moreover, for monetary and social rewards, cue letter B consistently evoked larger P3b amplitudes than cue letter A (*p* < 0.001). The analysis did not show a significant main effect of reward type [*F*_(1, 19)_ = 3.052, *p* = 0.097, *η*^2^*_p_* = 0.138]. However, a significant main effect of cue type [*F*_(1, 19)_ = 69.607, *p* < 0.001, *η*^2^*_p_* = 0.786] was noted, indicating that cue letter B evoked significantly larger P3b amplitudes than cue letter A (Table 3, Figure 6).

For the CNV components analysis, the reward type's main effect was insignificant [*F*_(1, 19)_ = 0.016, *p* = 0.900, *η*^2^*_p_* = 0.001]. However, a significant main effect of cue type was observed *F*_(1, 19)_ = 9.531, *p* = 0.006, *η*^2^*_p_* = 0.334), indicating that cue letter A induced significantly larger CNV amplitudes than cue letter B. The association between reward type and cue type was also nonsignificant [*F*_(1, 19)_ = 0.015, *p* = 0.905, *η*^2^*_p_* = 0.001]. Further analyses revealed that in the social rewards condition, cue letter A induced larger CNV amplitudes than cue letter B (*p* = 0.022) (Table 3, Figure 6).

## 4 Discussion

Highlighting the comparative impact of monetary vs. social rewards, our findings elucidate that social rewards significantly bolster proactive control in table tennis athletes under stress, marking a distinct advantage over monetary incentives. We report enhanced proactive control in response to social rewards, as evidenced by superior behavioral performance metrics and larger P3b amplitudes in ERP analyses, suggesting that social cues may exert a stronger effect on reward processing under stress.

Rewards are potent motivators capable of influencing an individual's cognitive control (73). Moreover, the positive effects associated with anticipating and receiving rewards can help alleviate the impact of acute stress (74). Regarding behavioral performance, we observed a higher PBI for both reaction time and error rate in response to monetary rewards than in the no-reward condition. This implies that monetary rewards, known as potent external motivators, can assist table tennis athletes in maintaining attention by inducing positive emotions and motivation. Consequently, this helps in reducing interference from distracting stimuli, ultimately enhancing reaction speed and accuracy in experimental tasks (75–77) and promoting proactive control (78). Inconsistent with the behavioral hypothesis, the enhancement of proactive control by social rewards was not observed in the behavioral index, despite being evident in the ERP results. This dissociation highlights a critical difference in sensitivity between electrophysiological and behavioral indices. Electrophysiological indices capture rapid, subtle modulations in attention and cognitive resource allocation, whereas overt behavioral improvements often require stronger or more sustained effects to attain statistical significance. And the efficacy of social rewards is highly contingent upon an athlete's subjective interpretation and prior experiences, leading to considerable individual variability. The standardized social cues used in this laboratory setting, while motivationally significant, lack the immediacy and personalization of real-world social interactions and the direct performance-contingent link of monetary incentives. These ecological constraints may have limited the translation of robust neural engagement into a significant, group-level behavioral advantage.

The reward anticipation phase involves the anticipation and desire for an impending or potential reward, serving as a motivating factor for the individual, with dopamine neuronal activity in the striatum playing a crucial role in the process (79). Under acute stress conditions, monetary and social rewards induced larger Cue-P2 and Cue-P3 amplitudes than no reward. Previous research has shown that reward-related cues such as color can enhance early cognitive processing stages, as indicated by increased P1 and N2pc components (80), promoting attention to task-relevant stimuli and reducing distraction from emotional cues (81). Our ERP results support the notion that both monetary and social rewards enhance attentional engagement, consistent with previous findings that reward-related cues promote early perceptual processing and sustained allocation of cognitive resources. The striatum is critical in integrating various types of reward stimuli into a common neural currency (82), and functional connectivity studies confirm that this universal pathway supports value assessment, motivational relevance, and action readiness during reward anticipation (83–85).

Aligned with cognitive disturbance and processing efficiency theories (86, 87), our results demonstrate that anticipation of monetary and social rewards mitigates stress-induced diversion of cognitive resources to negative affect. This mitigation is evidenced by enhanced P2 and P3b amplitudes, signifying bolstered cognitive control and processing efficiency under stress conditions (71, 72, 88). Such findings underscore rewards' pivotal role in sustaining athletes' performance amidst acute stress. A significant observation involves the CNV component, where the cue letter “A” under reward scenarios induced more pronounced negative CNV amplitudes than in the no-reward scenarios. This indicates that rewards enhance cognitive control by boosting anticipation and readiness for impending stimuli (89) and enhancing athletes' preparedness for actions. This heightened CNV response to anticipated stimuli underscores the role of rewards in optimizing cognitive control mechanisms.

This study focused on evaluating the unique effects of social rewards on athletes' proactive control under high-stress competitive conditions. Our observations revealed a notable enhancement in proactive control among table tennis athletes when subjected to social rewards conditions. Compared with that toward monetary rewards, the larger P3b amplitudes observed in response to social rewards may reflect the unique ability of social rewards to involve athletes' cognitive processing and resource allocation under stress. This could be due to their direct relevance to athletes' social and psychological needs. This advantage may be attributed to the ability of social rewards to fulfill fundamental psychological needs, such as social belonging and self-efficacy, fostering a profound stimulation of intrinsic motivation. This reinforcement of intrinsic motivation prompts athletes to proactively manage cognitive resources and optimize response preparation and execution when confronting stress and challenges. Furthermore, Ossewaarde et al. (90) suggested that acute stress diminishes striatal responses to monetary rewards, which potentially explains why social rewards may be more effective than monetary rewards in promoting proactive control. Our findings revealed no significant amplitude differences in the Cue-P2 and Cue-P3 components between reward types. This may indicate that both types of rewards primarily stimulate anticipation and motivation for the impending reward during the anticipation stage, indicating a commonality in the brain mechanisms underlying the anticipation of diverse rewards. Despite the differences in the nature of rewards, the activation of brain regions and electrophysiological responses during the primary motivational phase may exhibit similarities across reward types.

This study has some limitations. Given the emphasis on prioritizing ecological validity in competitive sports settings and the limited behavioral relevance of social rewards to non-athlete populations within athletic paradigms, this study abstained from including non-athlete participants, which may impose constraints on the generalizability of findings. Future studies are therefore recommended to include non-athlete samples or athletes from different sport levels as comparison groups to better evaluate the generalizability and boundary conditions of these findings. The use of the MAST stress paradigm may not fully replicate the stress experienced during actual training and competition, impacting the ecological validity of our findings. Future studies could improve ecological validity by using virtual sports simulations with VR technology to create more realistic and sports-specific stress-inducing scenarios.

In conclusion, our investigation highlights the differential impact of monetary and social rewards on enhancing proactive control in table tennis athletes under acute stress, as elucidated through ERP measurements. Notably, while both reward types were found to bolster cognitive control, social rewards demonstrated a distinct advantage in amplifying athletes' proactive responses, potentially owing to their alignment with intrinsic motivational factors and social-psychological needs. This insight not only advances our understanding of the motivational dynamics in sports but also suggests practical approaches for leveraging social incentives to optimize performance.

## Data Availability

The raw data supporting the conclusions of this article will be made available by the authors, without undue reservation.
