# Supplementary material for: Enhanced proactive control under stress: divergent neural dynamics of social vs. Monetary rewards in table tennis athletes
Source: Front Sports Act Living. 2025 Nov 19;7:1591411. doi: 10.3389/fspor.2025.1591411 (PMC12672868; doi:10.3389/fspor.2025.1591411)
Supplement: Supplementary file 1 [file Supplementaryfile1.docx]

**Acute Stress Manipulation Examination**

To systematically examine the effects of acute stress, our experimental procedure was structured around five key time points: baseline (t1), immediately following the first acute stress induction (t2), after completing the first AX-CPT task (t3), subsequent to the second acute stress induction (t4), and following the second AX-CPT task (t5). This sequence was designed to assess the dynamics of stress response and recovery.

To verify the efficacy of our acute stress manipulations, we conducted manipulation checks, analyzing both heart rate and self-reported stress levels across these time points. These checks were performed utilizing IBM SPSS Statistics for Windows, version 27.0. A repeated-measures ANOVA was applied to the data collected at t1, t2, t3, t4, and t5. In instances where the assumption of sphericity was not met, we resorted to multivariate analyses. To ensure the integrity of our findings, adjustments for multiple comparisons were made using the LSD method, focusing on all main effects observed.

***Monetary reward***

(1) Heart rate

In the analysis of main effects across time points, significant variations in heart rate were observed (*F*_(4, 19)_ = 19.369, *p* < 0.001, *η^2^_p_* = 0.803). Specifically, heart rate at time point t2 was significantly higher than at t1, t3, and t5 (all p < 0.001), but not significantly different from t4 (*p* = 0.068). Heart rate at t3 was significantly elevated compared to t1 (*p* = 0.021) and showed a significant decrease from t2 and t4 (both *p* < 0.001), with no significant difference from t5 (*p* = 0.808). Following the second stress manipulation, heart rate at t4 was significantly higher compared to t1, t3, and t5 (all *p* < 0.001), but not significantly different from t2 (*p* = 0.068). These findings highlight significant changes in heart rate following stress manipulations, reflecting dynamic adjustments in heart rate across different time points (Table 1).

(2) Self-reported stress

In assessing the primary effects of time points on self-reported stress scores, a significant variation was noted (*F*_(4, 88)_ = 18.848, *p* < 0.001, *η^2^_p_* = 0.461), indicating fluctuating stress levels among table tennis athletes at different assessed points. Specifically, athletes reported the highest stress scores at the t2 time point, which were significantly higher than the initial t1 (*p* < 0.001), and showed a marked decrease by t3 (*p* = 0.014) and further by t5 (*p* = 0.008), although no significant difference was observed when compared to t4 (*p* = 0.451). Stress scores at t3, while significantly higher than at t1 (*p* < 0.001), were notably less than those observed immediately after the stressor at t2 (*p* = 0.014) and showed no significant difference from t5 (*p* = 0.628), indicating a temporary reduction in perceived stress. The subsequent assessment at t4 again showed elevated stress scores, comparable to t2 and significantly higher than both the initial t1 (*p* < 0.001) and the reduced levels at t3 (*p* = 0.007) and t5 (*p* = 0.015), suggesting a resurgence in stress perception. By the final t5 time point, stress scores had decreased significantly from their peak at t2 (*p* = 0.008) but remained above the baseline established at t1 (*p* < 0.001), reflecting an ongoing adaptation to the stressor (Table 1).

**Table 1.** Descriptive statistics of heart rate and self-reported stress in table tennis athletes at 5 time points in the monetary reward AX-CPT task (*M* ± *SD*).

| **time point** | **t1** | **t2** | **t3** | **t4** | **t5** |
| --- | --- | --- | --- | --- | --- |
| **heart rate** | 68.056 ± 6.668 | 86.385 ± 9.901 | 69.277 ± 6.294 | 84.123 ± 10.396 | 69.441 ± 7.262 |
| **self-reported stress** | 1.696 ± 0.635 | 3.261 ± 0.864 | 2.522 ± 0.947 | 3.130 ± 0.757 | 2.609 ± 0.941 |

***Social reward***

(1) Heart rate

Significant main effects for time point changes in the heart rate of table tennis athletes were identified (*F*_(4, 17)_ = 35.450, *p* < 0.001, *η^2^_p_* = 0.893). Post hoc analyses revealed that heart rate was highest at t2, immediately following the acute stress manipulation, with significantly elevated levels compared to all other time points. Specifically, at t3, there was a significant reduction in heart rate from its peak at t2 (*p* < 0.001), though it remained higher than the baseline measured at t1 (*p* < 0.001). Notably, no significant difference was seen between t3 and t5 (*p* = 0.673). After the second acute stressor, heart rate at t4 was significantly higher than at t1, t3 and t5 (all *p* < 0.001), indicating a stress response similar to t2, albeit with a slight reduction (*p* = 0.002) (Table 2).

(2) Self-reported stress

The analysis revealed a significant main effect for time points on self-reported stress scores (*F*_(4, 80)_ = 23.077, *p* < 0.001, *η^2^_p_* = 0.536). Following the acute stress induction, stress scores at t2 were significantly elevated compared to baseline t1 (*p* < 0.001) and showed significant reductions at t3 (*p* = 0.038) and t5 (*p* = 0.009). While the scores at t3 were still significantly higher than t1 (*p* < 0.001), they were lower than at the peak observed at t2 (*p* = 0.038), and there was no significant difference from the scores at t5 (*p* = 0.480), suggesting a stabilization of stress. The highest self-reported stress score was recorded at t4, significantly higher than at t1 (*p* < 0.001), t3 (*p* = 0.008), and t5 (*p* < 0.001), but it did not differ significantly from the earlier peak at t2 (*p* = 0.358), indicating a second stress response plateau (Table 2).

**Table 2.** Descriptive statistics of heart rate, stress self-report and negative affect in table tennis athletes at 5 time points in the social reward AX-CPT task (*M* ± *SD*).

| **time point** | **t1** | **t2** | **t3** | **t4** | **t5** |
| --- | --- | --- | --- | --- | --- |
| **heart rate** | 66.600 ± 8.147 | 88.241 ± 15.408 | 70.291 ± 10.047 | 83.201 ± 12.996 | 69.956 ± 9.474 |
| **self-reported stress** | 1.476 ± 0.680 | 3.334 ± 0.913 | 2.810 ± 0.814 | 3.524 ± 0.814 | 2.667 ± 0.856 |
